# Malnutrition as a major related factor of frailty among older adults residing in long-term care facilities in Korea

**DOI:** 10.1371/journal.pone.0283596

**Published:** 2023-04-07

**Authors:** SeolHwa Moon, Eunmi Oh, Daum Chung, Rina Choi, Gwi-Ryung Son Hong

**Affiliations:** 1 Department of Nursing, Hoseo University, Cheonan-si, South Korea; 2 Research Institute of Nursing Science, Hanyang University, Seoul, South Korea; 3 School of Nursing, Hanyang University, Seoul, South Korea; NIPSOM: National Institute of Preventive and Social Medicine, BANGLADESH

## Abstract

**Objectives:**

The objectives of this study were 1) to investigate the prevalence and co-existence of frailty and malnutrition and 2) to identify factors related to frailty (including malnutrition) according to the level of frailty.

**Methods:**

Data collection was conducted from July 11, 2021, to January 23, 2022, in 558 older adults residing in 16 long-term care facilities (LTCFs) in Korea. The FRAIL-NH and Mini-Nutritional Assessment short form were used to measure frailty and nutrition, respectively. The data analysis included descriptive statistics and a multivariate logistic regression.

**Results:**

The mean age of the participants was 83.68 (± 7.39) years. Among 558 participants, 37 (6.6%), 274 (49.1%), and 247 (44.3%) were robust, prefrail, and frail, respectively. At the same time, 75.8% were categorized as having malnutrition status (malnourished: 18.1%; risk of malnutrition: 57.7%), and 40.9% had co-existing malnutrition and frailty. In the multivariate analysis, malnutrition was identified as the major frailty-related factor. Compared with a normal nutritional status, the incidence of frailty in the malnutrition group was 10.35 times (95% CI: 3.78–28.36) higher than the incidence of robustness and 4.80 times (95% CI: 2.69–8.59) higher than the incidence of prefrail.

**Conclusion:**

The prevalence of frailty and malnutrition, and their co-existence, among older adults residing in LTCFs was high. Malnutrition is a major factor that increases the incidence of frailty. Therefore, active interventions are needed to improve the nutritional status of this population.

## Introduction

Frailty is one of the biggest problems facing the older population and is defined as a reduction in reserve capacity to resist mild stressors, an increase in vulnerability to unhealthy consequences, and damage to various physiological systems [1]. The increased vulnerability seen with frailty is a risk factor for outcomes such as falls, disability, hospitalization, and death [2]. These results cannot be overlooked because they increase the demand for medical and social welfare services in the older population and ultimately increase social costs [2].

The prevalence of frailty in long-term care facilities (LTCFs) has been reported to be 25.9–68.8% [3–5], which is higher than the community incidence of 19.1% [6]. The high variation in the prevalence of frailty in LTCFs might be due to differing definitions of frailty and the use of different measurement scales [4].

Frailty is related to demographic factors, health-related factors, physical factors, psychological factors, nutritional factors, and sarcopenia [7]. Malnutrition has been reported to be closely related to frailty. The anorexia of aging leads to chronic malnutrition and increases dependency and sarcopenia, which is a decrease in muscle mass, and the starting point in the cycle of frailty [2]. Malnutrition is a well-known factor in the development of frailty, and it co-exists with frailty at a high incidence rate [6–8]. A meta-analysis of older adults in a community reported that 68% of the malnourished participants were frail, and 10% of the frail older adults were malnourished or at risk of malnutrition [6]. Despite the high association between frailty and nutrition, most studies have considered only older community-dwelling adults, and few have investigated the relationship between the two factors in LTCFs. In addition, because the older adults in LTCFs are typically more vulnerable in their daily functioning than those in the community, it is necessary to identify the factors related to frailty (including malnutrition) in this population to recognize any differences from community dwellers.

Although the lack of an evidence-based treatment standard makes it difficult to prevent and manage frailty [1], it is necessary to at least control the occurrence of frailty by identifying modifiable risk factors. Considering the multidimensional and heterogeneous characteristics of senescence and the complex needs of frail older adults [4], identifying frailty-related factors will help researchers and clinicians develop effective intervention methods to prevent frailty among LTCF residents. Therefore, the purposes of this study were as follows; 1) to investigate the prevalence of frailty and malnutrition and their co-existence and 2) to identify the factors related to frailty, including malnutrition, according to the level of frailty.

## Method

### Study design and sample (population)

Data were obtained from an ongoing longitudinal study by the corresponding author of this paper starting in July 2020 to assess the risk factors for mortality in older adults residing in LTCFs. This preliminary data analysis was conducted using cross-sectional data collected in the first year of the ongoing longitudinal study to identify the frailty-related factors in older adults who reside in LTCFs. The sample size for this secondary data analysis study was calculated with G*Power (Version 3.1.9.2, Franz Faul, University Kiel, Kiel, Germany) using the following values for the logistic regression: odds ratio (effect size) of 1.70 [9], a study power of 95%, and an alpha of 0.05. The minimum required sample size for this study was 170.

To secure the representativeness of the data, the original study planned to draw its population from eight of the 17 provinces in South Korea with 5.0% or more of their residents in LTCFs according to the “2018 Status Welfare Facilities for Older Adults” [10]. However, due to the effects of coronavirus disease-19 (COVID-19) pandemic, the study process was delayed, and recruiting potential LTCFs became difficult. Therefore, the original study was conducted in 16 LTCFs in six provinces (Seoul, Gyeonggi, Chungcheong, Daejeon, Incheon, and Gyeongsang provinces). The inclusion criteria for this study were people older than 65 years who had resided in an LTCF for more than two months, had the ability to communicate, and agreed to participate in the study. The exclusion criteria were an inability to communicate and residing in an LTCF for less than two months.

### Data collection

At the time of data collection, access to the LTCFs was restricted due to the COVID-19 pandemic; therefore, all measurements were conducted by the staff of the LTCFs. The 27 staff members who administered the measurements were registered nurses or social workers employed at the participating LTCFs. They registered on the online system developed by the research team to review about 120 minutes of video clips in seven videos describing the methods and measurements for all instruments. The videos for the FRAIL-NH and MNA-SF scales, explained the detailed methods for each item and precautions. The video for anthropometric measurements, explained the instruments and demonstrated how to take the measurements on an actual participant. For example, calf circumference was first shown using a plastic measuring tape. Next, the posture of the participants (sitting or supine position with knee flexion at 90 degrees), the point of measurement (the thickest part in the middle calf), and procedure for reporting the results (the average value of both calves) were demonstrated and explained step by step. In addition, a study manual containing detailed descriptions and figures was provided to minimize inter-rater errors. Research assistants were available by phone whenever LTCF staffs needed help or had questions. The online system was available only for authorized LTCF staff members, and the research team monitored their learning progress through an online database. After they had watched all the video clips, they were allowed to start the actual data collection process. Measurement tools and questionnaires were sent to the LTCF staffs about two weeks before data collection, so they could familiarize themselves with the measurement tools. The research assistant re-guided the overall process of the survey and answered all additional questions related to the measurement. Prior to collecting data, the LTCF staff explained the purpose of the study to potential participants and asked whether they were interested in participating in the study. Written informed consent was obtained after they agreed, and they were told that they could withdraw from the study at any time. The staff members completed the questionnaires by reviewing medical records, conducting face-to-face interviews, taking body measurements, and observing the participants as specified by the questions.

### Measurements

#### Nutritional status

Nutritional status was evaluated using the MNA-SF, which consists of six items about appetite loss, weight loss, mobility, stress/acute illness, dementia/depression, and body mass index (BMI). BMI is calculated using the patients’ height and weight. Participants who had difficulty being measured for height and weight because they used a wheelchair or were bedridden were instead assessed using calf circumference, as specified in the MNA-SF guidelines [11]. The maximum score was 14 points, and nutritional status was classified into the following three groups: 12–14, normal nutrition; 8–11, at risk of malnutrition; 0–7, malnourished [12]. According to the purpose of this study, nutritional status was further classified into two groups: normal (12–14) and malnutrition (0–11) [9].

#### Frailty

Frailty was evaluated using version 2 of the FRAIL-NH scale [13]. In version 2, “I” represents illness instead of incontinence. The FRAIL-NH (version 2) consists of seven items: F (fatigue), R (resistance), A (ambulation), I (illness), L (loss of weight), N (nutritional approach), and H (help with dressing). Each item is rated on a 3-point Likert scale (0–2), so the total score ranges from 0–14, with a higher score indicating greater frailty. In this study, the total score was categorized as robust (0–1), prefrail (2–5), and frail (6–14) [5, 14]. Only participants with valid scores in 6–7 of the 7 FRAIL-NH items were included in the analyses in this study [5].

#### Covariates

Data about hypertension, heart disease, stroke, comorbidity, urinary incontinence (UI), depressive symptom, and dry mouth among the participants were collected as health-related factors. Hypertension, heart disease, stroke, and other comorbidities were included if they appeared in the medical chart review. Comorbidities were calculated as the number of diseases of the following diseases: hypertension, diabetes mellitus, lung disease, cataracts, arthritis, UI, osteoporosis, Parkinson’s disease, heart disease, stroke, cancer, and dementia. UI was assessed by asking the nursing staff about the presence of UI symptoms. The presence of depressive symptoms was assessed by asking the staff to answer the question, “Does the patient look sad or depressed?”, which is one of the questions in the Korean version of the Neuropsychiatric Inventory [15]. Possible answers were “yes” and “no.” Dry mouth was measured by asking the participants: “How often does your mouth feel dry?”, and asking them to choose one of the following options: “Never,” “Occasionally,” “Frequently,” or “Always” [16]. In this study, “Never” and “Occasionally” were reclassified as no dry mouth and “Frequently” and “Always” were classified as dry mouth [17]. Grip strength was measured using a digital hand dynamometer (LAVISEN ks-301, Korea, kg). Both hands were alternatively measured twice with the patient in a comfortable sitting position. In older adults with hemiplegia, only the arm on the non-affected side was measured. Participants were asked to squeeze the handle as hard as possible, and the mean of the values (kg) measured from the right and left hands was used for analysis [18].

### Data analysis

The data analysis was conducted using IBM SPSS, version 23.0 (IBM Corp., Armonk, NY, USA) software. Differences in variables according to group (robust, prefrail, and frail) were analyzed with the chi-square (*x*^2^) test. The frequency of frailty according to nutritional status and the ratio of frailty and malnutrition were calculated using Excel. A univariate analysis identified significant factors according to the level of frailty (robust vs. prefrail: UI; robust vs. frail: stroke, comorbidities, UI, dry mouth, grip strength, nutritional status; prefrail vs. frail: sex, stroke, comorbidities, UI, dry mouth, grip strength, nutritional status). The multivariate logistic regression analysis used the backward method with variables that were significant in the univariate logistic analysis to identify factors related to different levels of frailty. The strength of those relationships was estimated as odds ratios with 95% confidence intervals. For statistical significance, *p* < 0.05 was considered.

## Results

**Fig 1:**
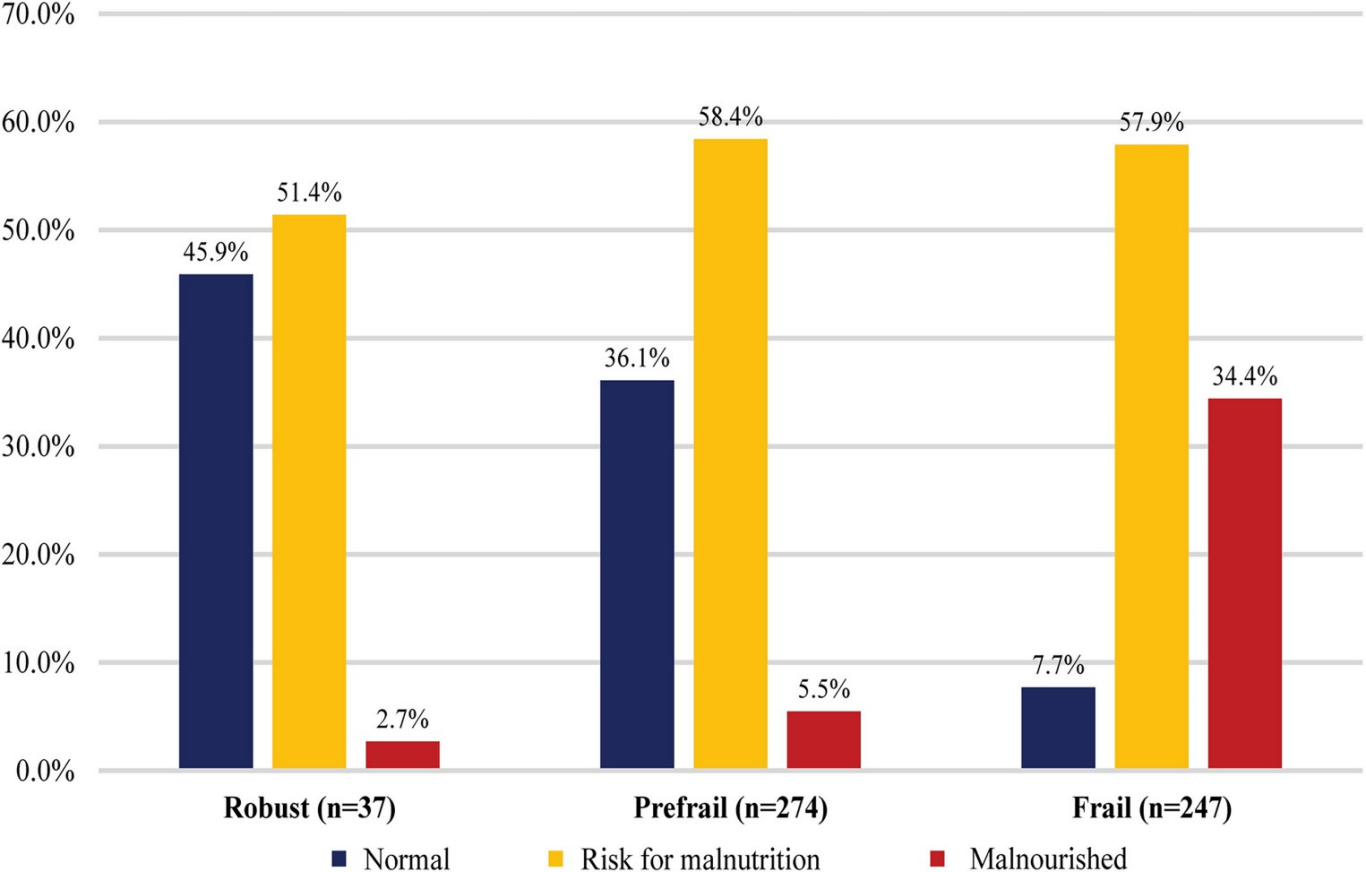
Prevalence of malnutrition according to frailty group.

**Fig 2:**
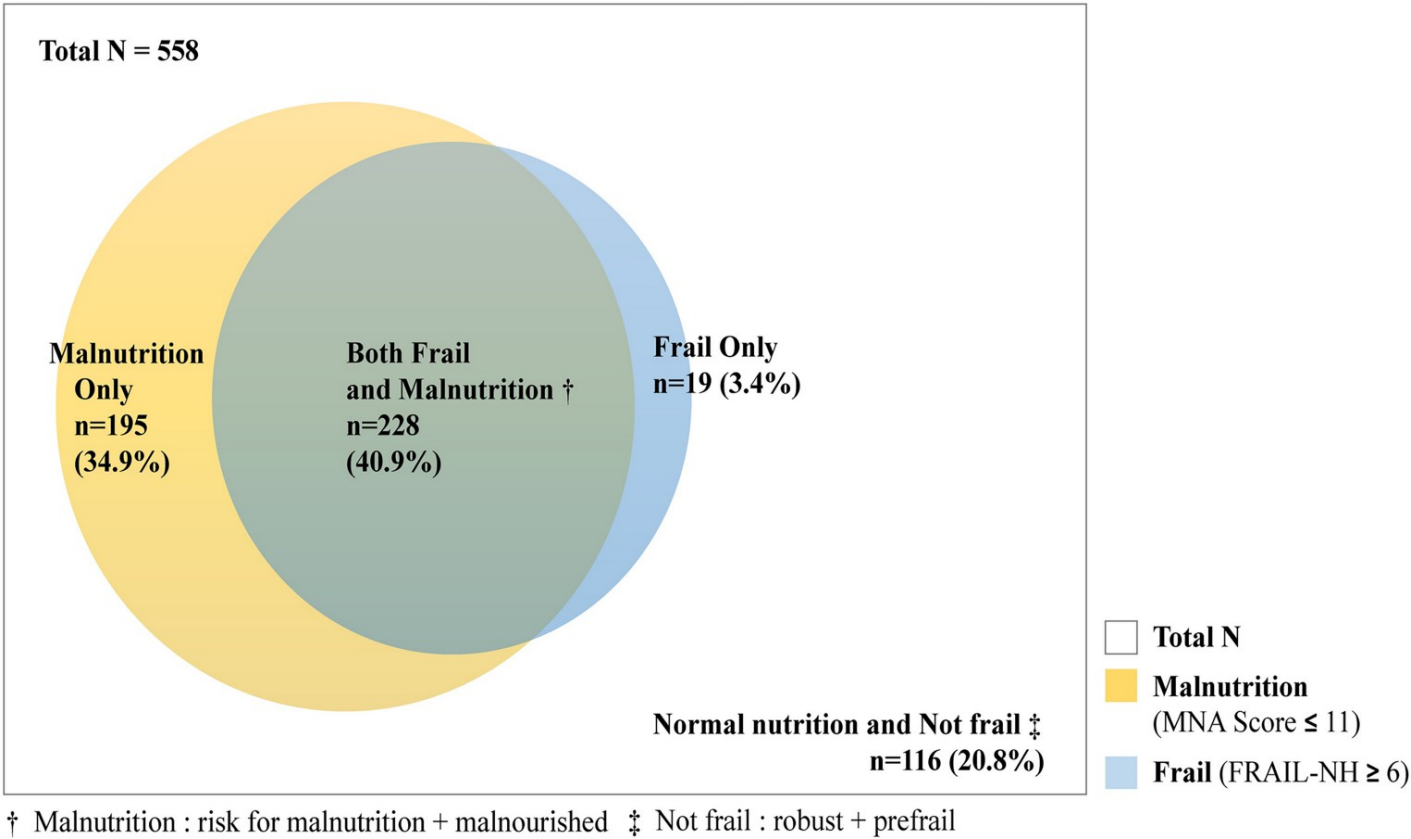
Venn diagram showing the co-existence of frailty and malnutrition.

This study included 558 participants, and their mean age was 83.68 ± 7.39 years. The participants’ characteristics and differences in variables according to frailty status are shown in Table 1. The numbers of participants in the robust, prefrail, and frail groups were 37 (6.6%), 274 (49.1%), and 247 (44.3%), respectively. The variables with statistically significant differences according to frailty status were sex (*p* = .037), stroke (*p* = .002), comorbidity (*p* = .006), UI (*p <* .001), dry mouth (*p <* .001), grip strength (*p <* .001), and nutritional status (*p <* .001). The prevalence rates of malnourishment according to frailty group were 2.7% in the robust group, 5.5% in the prefrail group, and 34.4% in the frail group (Fig 1). The co-existing prevalence rates of frailty and malnutrition (risk of malnutrition and malnourished) are presented in Fig 2. In all, 228 (40.9%) participants were classified as having both frail and malnourished status, and the groups with only malnutrition or frailty numbered 195 (34.9%) and 19 (3.4%), respectively (Fig 2).

**Table 1:** Characteristics of the participants and differences in the variables among frailty groups (N = 558).

Variables	Total	Robust	Prefrail	Frail	t or *x*^2^ (*p*)
	(n = 558)	(n = 37)	(n = 274)	(n = 247)	
	M ± SD or	M ± SD or	M ± SD or	M ± SD or	
	n (%)	n (%)	n (%)	n (%)	
Age (year)	83.68 ± 7.39	84.43 ± 7.91	83.31 ± 7.38	83.98 ± 7.33	0.75 (.475)
Sex					
Female	435 (78.0)	27 (73.0)	203 (74.1)	205 (83.0)	6.57 (.037)
Male	123 (22.0)	10 (27.0)	71 (25.9)	42 (17.0)	
Hypertension					
Yes	178 (31.9)	7 (18.9)	84 (30.7)	87 (35.2)	4.32 (.115)
No	380 (68.1)	30 (81.1)	190 (69.3)	160 (64.8)	
Heart disease				
Yes	58 (10.4)	2 (5.4)	30 (10.9)	26 (10.5)	1.08 (.582)
No	500 (89.6)	35 (94.6)	244 (89.1)	221 (89.5)	
Stroke						
Yes	96 (17.2)	2 (5.4)	37 (13.5)	57 (23.1)	12.23 (.002)
No	462 (82.8)	35 (94.6)	237 (86.5)	190 (76.9)	
Comorbidities	3.24 ± 1.58	2.68 ± 1.42	3.13 ± 1.46	3.44 ± 1.71	5.10 (.006)
UI					
Yes	343 (61.5)	10 (27.0)	145 (52.9)	188 (76.1)	50.46 (< .001)
No	211 (37.8)	27 (73.0)	127 (46.4)	57 (23.1)	
Missing	4 (0.7)		2 (0.7)	2 (0.8)	
Depressive symptoms				
Yes	272 (48.8)	16 (43.2)	138 (50.4)	118 (47.8)	0.79 (.673)
No	282 (50.5)	21 (56.8)	135 (49.3)	126 (51.0)	
Missing	4 (0.7)		1 (0.3)	3 (1.2)	
Dry mouth						
Yes	98 (17.6)	2 (5.4)	239 (87.2)	174 (70.4)	28.84 (< .001)
No	447 (80.1)	34 (91.9)	29 (10.6)	67 (27.1)	
Missing	13 (2.3)	1 (2.7)	6 (2.2)	6 (2.4)	
Grip strength (kg)	11.80 ± 8.22	14.62 ± 8.27	13.20 ± 8.16	9.57 ± 7.78	14.41 (< .001)
MNA (score)	9.66 ± 2.24	11.16 ± 1.61	10.54 ± 1.84	8.45 ± 2.14	85.20 (< .001)
Malnourished	101 (18.1)	1 (2.7)	15 (5.5)	85 (34.4)	116.75 (< .001)
Risk of malnutrition	322 (57.7)	19 (51.4)	160 (58.4)	143 (57.9)	
Normal	135 (24.2)	17 (45.9)	99 (36.1)	19 (7.7)	

UI: urinary incontinence; MNA: Mini Nutritional Assessment

The results of the univariate analysis are presented in Table 2. The only factor significantly related to prefrail status (reference: robust) was UI. The factors significantly related to frail status (reference: robust and prefrail) were stroke, comorbidities, UI, dry mouth, grip strength, and nutritional status. The only factor significantly related to frail status (reference: prefrail) was being female.

**Table 2:** Univariate logistic regression models for frailty (N = 558).

Variables	Robust vs. Prefrail	Robust vs. Frail	Prefrail vs. Frail
	OR (95% CI)	OR (95% CI)	OR (95% CI)
Age (years)	0.98 (0.93–1.03)	0.99 (0.95–1.04)	1.01 (0.99–1.04)
Sex			
Female	1.06 (0.49–2.30)	1.81 (0.81–4.02)	1.71 (1.11–2.62)*
Male (ref)			
Hypertension			
Yes	1.89 (0.80–4.49)	2.33 (0.98–5.52)	1.23 (0.85–1.77)
No (ref)			
Heart disease			
Yes	2.15 (0.49–9.40)	2.06 (0.47–9.06)	0.550.96 (0.55–1.67)
No (ref)			
Stroke			
Yes	2.73 (0.63–11.84)	5.25 (1.23–22.50)*	1.92 (1.22–3.03)**
No (ref)			
Comorbidities	1.27 (0.97–1.65)	1.39 (1.08–1.79)*	1.13 (1.02–1.27)*
UI			
Yes	3.08 (1.44–6.62)**	8.91 (4.07–19.50)***	2.89 (1.98–4.23)***
No (ref)			
Depression			
Yes	1.34 (0.67–2.68)	1.23 (0.61–2.47)	0.92 (0.65–1.29)
No (ref)			
Dry mouth			
Yes	2.06 (0.47–9.04)	6.55 (1.53–28.01)*	3.17 (1.97–5.12)***
No (ref)			
Grip strength (kg)	0.98 (0.94–1.02)	0.94 (0.90–0.98)**	0.94 (0.92–0.96)***
Nutritional status			
Malnutrition	1.50 (0.75–3.00)	10.20 (4.59–22.66)***	6.79 (4.00–22.52)***
Normal (ref)			

*p < .05

** *p* < .01

*** *p* < .001; OR: odds ratio

CI: confidence interval

ref: reference

UI: urinary incontinence

The results of the multivariate analysis are presented in Table 3. Compared with a normal nutritional status, the incidence of frailty in participants with malnutrition were 10.4 times (OR: 10.35, 95% CI: 3.78–28.36) higher than the incidence of robustness and 4.8 times (OR: 4.80, 95% CI: 2.69–8.59) higher than the incidence of prefrailty. Prefrail status did not differ significantly from robust status in the incidence of malnutrition. The following variables were also significantly different with an increased incidence of frailty according to reference group: 1) reference group in robust status: UI (OR: 8.25, 95% CI: 3.19–21.34), comorbidities (OR: 1.67, 95% CI: 1.22–2.30); 2) reference group in prefrail status: UI (OR 2.05, 95% CI: 1.31–3.22), comorbidities (OR 1.31, 95% CI 1.15–1.51), dry mouth (OR: 2.96, 95% CI: 1.73–5.08), and grip strength (OR: 0.95, 95% CI: 0.92–0.98). UI (OR: 3.08, 95% CI: 1.44–6.62) was the only factor associated with prefrailty (reference: robust).

**Table 3:** Multivariate logistic regression models for frailty (N = 558).

Variables	Robust vs. Prefrail	Robust vs. Frail	Prefrail vs. Frail
	OR (95% CI)	OR (95% CI)	OR (95% CI)
UI			
Yes	3.08 (1.44–6.62)**	8.25 (3.19–21.34)***	2.05 (1.31–3.22)**
No (ref)			
Comorbidities		1.67 (1.22–2.30)**	1.31 (1.15–1.51)***
Dry mouth			
Yes			2.96 (1.73–5.08)***
No (ref)			
Grip strength (kg)			0.95 (0.92–0.98)**
Nutritional status			
Malnutrition		10.35 (3.78–28.36)***	4.80 (2.69–8.59)***
Normal (ref)			
Constant	4.70***	0.06***	0.09***
	Model summary: Nagelkerke R^2^ = 0.057	Hosmer-Lemeshow test: *x*^*2*^ = 6.69 *df* = 6, *p* = .350	Hosmer-Lemeshow test: *x*^*2*^ = 7.78, *df* = 8, *p* = .455
		Model summary: Nagelkerke R^2^ = 0.452	Model summary: Nagelkerke R^2^ = 0.275

*p < .05

** *p* < .01

*** *p* < .001

ref: reference

OR: odds ratio

CI: confidence interval

UI: urinary incontinence

## Discussion

In this study, the prevalence rates of prefrailty and frailty were 49.1% and 44.3%, respectively, with only 6.6% of participants classified as robust. Although that prevalence of frailty is different from that reported in previous studies (25.9–68.8%) [3–5], it is similar to the results (47.4–54.2%) of previous studies that used the same instrument (FRAIL-NH) [3, 5]. In a previous study that identified the level of frailty among the older adults residing in long-term care hospital (LTCH) in Korea, 49.0% were found to be frail (FRAIL-NH>10) [19]. However, the previous study was a retrospective review of frailty among participants who died in the LTCH, so it can be assumed that they were more frail than the participants of this study.

The prevalence of frailty in this study is higher than the 25.9% reported among Korean older adults living in the community [20], which supports the results of previous studies [3, 5, 6]. The prevalence of dry mouth in this study was 17.6%, which is similar to a previous study [21]. Oral health is closely related to frailty, with frail older adults having a higher rate of complaint about dry mouth than robust older people. The results of this study are similar to those of previous studies that reported that frail older adults had poorer oral health (e.g., chewing difficulty, general oral health) than robust older adults [20, 22]. The prevalence of malnutrition (including those malnourished and at risk for malnutrition) in this study was 75.8%. Previous studies targeting the residents of care homes reported a prevalence of malnutrition of 42.9%–87.7%, depending on the measurement scales used [23, 24]. Other studies that assessed the nutritional status of LTCF residents using the MNA-SF reported malnutrition rates of 60.7–85.4% [25, 26], which is similar to the findings of this study. Reasons for the large variation in the prevalence of frailty and malnutrition could be LTCFs of different sizes, the heterogeneity of participant characteristics, or the use of different measurement scales [4, 27]. The MNA-SF is valid and reliable among Asian older population residing in clinics and the community [11], and this instrument has been widely used for Korean older adults [9, 28]. The FRAIL-NH also has established validity and reliability among the residents of LTCFs in Korea [19]. Furthermore, nutritional status and frailty measured using those scales are reported to predict negative outcomes in this population [13, 29]. Both instruments contain only six or seven uncomplicated items, so they are easy to administer [30]. Moreover, the FRAIL-NH is a simpler instrument for assessing the overall frailty of LTCF residents than other frailty measurement tools [30]. However, it is necessary to interpret these results with caution because the characteristics that overlap between the items in the FRAIL-NH and the MNA-SF (weight loss, decreased physical function, and psychological symptoms) could overestimate the relationship between frailty and malnutrition [31]. In this regard, Soysal and colleague reported a strong relationship between each item of the MNA-SF and the FRAIL-NH, and high sensitivity and specificity for detecting frailty [32]. In other words, the high correlation between the two instruments makes it necessary to be careful in interpreting the results of this study [32].

The high prevalence of frailty and malnutrition in older adults residing in LTCFs not only increases their rates of hospitalization, mortality, and morbidity, but also is a high risk factor for mortality when those two factors co-exist [33]. In this study, frailty and malnutrition co-existed in 288 patients (40.9%), which is similar to the findings of previous studies, which reported a 33.5–40.5% incidence of co-existing frailty and malnutrition [25, 34]. Given that the prevalence rate of frailty and malnutrition among community-dwelling older adults is about 10% [6], our finding clearly reflects the high vulnerability of LTCF residents. Progressive malnutrition is influenced by multiple factors, and shortens lifespans, as well as increasing morbidity and length of hospitalizations among older adults [35]. In addition, frailty syndrome, a state that decreases a person’s resistance to stress, increases care needs, and admission to hospitals or LTCFs [36]. The coexistence of frailty and malnutrition is thus a strong predictor of poor outcomes such as increased medical costs, morbidity, and mortality [33, 34]. Sharma and colleagues reported that the coexistence of frailty and malnutrition worsens clinical outcomes and lengthens hospital stays [34]. Another study reported that the coexistence of these two factors is associated with a greater all-cause mortality (HR = 10.89) among LTCF residents [33]. Careful observation and assessment of these two factors are required through periodic screening test, and prompt intervention is needed to minimize the occurrence of fatal results.

Malnutrition and frailty can be modified using an appropriate strategic approach [35]. A previous study reported that the application of a multicomponent intervention (e.g., nutritional supplements, group exercise, etc.) for 24 months among frail older adults in the community not only improved frailty but also lengthened lifespans and reduced institutionalization [37]. Because the frailty level among residents in LTCFs showed a dynamic change over a short period of two years [3], it is necessary to identify any related factors and then carry out a management strategy tailored to the frailty status of each individual. In other words, to improve malnutrition and frailty in the older adults residing in LTCFs, systematic and strategic interventions that consider the characteristics of both the residents and the LTCF setting will be needed.

In this study, malnutrition was identified as the major factor related to frailty. Several studies have consistently reported that malnutrition as measured by the MNA (or MNA-SF) is a major factor in frailty across differences in residential environment and measurement scale [31, 38] and this has been consistent not only in older adults in the community (OR = 17.4) [31] and in those in LTCFs (OR = 2.66) [26]. Nevertheless, several studies have confirmed that malnutrition as determined by the MNA-SF is a major factor related with frailty, and these consistent results are also reported in older adults residing in LTCFs [6, 31, 38]. Malnutrition in older adults is complex and multifactorial, and closely related to the anorexia of aging [39]. Among the lifestyle factors that affect the progression of frailty, malnutrition is classified as a modifiable factor [7]. Interventions to improve lifestyle have been reported to be an easy and effective method for improving patient nutritional status as well as improving and delaying the progression of frailty [40]. Nursing staff at LTCFs should practice careful observation and regular assessment along with active planning and interventions to improve the nutritional status of LTCF residents.

In this study, UI was identified as a factor that worsened all levels of frailty. UI causes various negative consequences, such as falls, skin complications, psychosocial limitations, and lower quality of life [41]. These consequences can accelerate the progression of frailty [42]. UI can be treated or managed successfully through appropriate interventions by nursing staff [41]. Nursing staff need evidence-based clinical skills and knowledge to conduct active evaluations and interventions to improve UI symptoms in older adults. The active engagement of nursing staff can prevent the deterioration of frailty caused by UI complications.

Chronic disease, dry mouth, and grip strength were also identified as factors related to frailty. These findings are consistent with the results of previous studies [7, 43]. A systematic review reported that oral health is closely related to frailty [43], and similar results have been reported in studies of Korean older adults [20, 22]. Lim and colleague reported that poor oral health status increased the risk of frailty and various geriatric syndromes [22]. Another study not only reported that perceived chewing difficulty increased the incidence of frailty, but also that frailty is a predictor of poor oral health [20]. In other words, poor oral health is both a result and driving force in the vicious cycle of frailty progression [20], and can be a major cause of poor outcomes. In this study, dry mouth was identified as the second most important factor in frailty. Dry mouth affects oral health and oral functions such as chewing ability and swallowing [43], which in turn affects the selection of foods, often leading to malnutrition [44]. These processes eventually affect the development of frailty. Because both oral health and malnutrition are linked to frailty, maintaining and increasing oral health is also effective in improving nutritional status and preventing frailty [44]. To prevent the occurrence of malnutrition and frailty according to poor oral health, it will be necessary to identify the factors related to dry mouth and conduct appropriate interventions in this population. Grip strength is also consistently reported as a factor related to frailty [45]. Since grip strength is an easier, quicker, and more applicable screening method for clinical sites, regular grip checks will help with early screenings for the progression of frailty.

This study found a difference in factors that correlated with prefrail and frail participants. Among prefrail people (reference: robust), only UI was a significantly related factor. Three factors were related to frailty (UI, comorbidities, and nutritional status) in reference to robust patients, and five factors (UI, comorbidities, dry mouth, grip strength, and nutritional status) were related to prefrailty. Because the related factors that can aggravate frailty differ according to the level of frailty in LTCF residents, an individual intervention approach that reflects the frail characteristics of each participant is required.

This study has a few limitations. First, although the participants were recruited from multiple centers (16 LTCFs distributed throughout six administrative districts in Korea), the generalizability of these findings to all Korean LTCFs is limited. Nationwide data collection was planned to secure the representativeness of the results, but difficulty in conducting data collection during the COVID-19 pandemic inevitably changed that plan. In a further study, it will be necessary to derive generalizable results by accurately representing this population in a cohort that reflects the regional distribution ratio of LTCFs in Korea. Second, the present study might not reflect the overall characteristics of the older adults residing in LTCFs because those with difficulty in communication were excluded. Third, the limitation of grip strength measurement caused by the influence of dominant arm hemiplegia might have affected the accuracy of that result. Fourth, the co-existing prevalence of frailty and malnutrition was high, but related factors or adverse effects of coexistence were not confirmed. Therefore, a longitudinal study is necessary to identify the causal relationship between frailty and its related factors and the co-existence effect of frailty and malnutrition among older adults who reside in LTCFs. Fifth, the interpretation of these results should consider the effects of the COVID-19 pandemic. Twenty-seven staff members participated in all measurements to control outsider access to the LTCFs. Although training and manuals were provided, inter-rater reliability might nonetheless have been compromised. In addition, the COVID-19 pandemic itself might have affected frailty among institutionalized older adults, because frailty is influenced by psychological factors such as depression, anxiety, and cognitive function. Isolation during the COVID-19 pandemic (prohibition of visits from family members and participation in various internal and external activity programs) must also have negatively affected physical function as well as psychological health [46]. It cannot be ruled out that the psychological distress exacerbated by the pandemic affected the frailty of this population.

## Conclusion

The incidence of frailty and malnutrition, as well as the co-existence of those two factors, was high among residents of LTCFs in Korea. Malnutrition was identified as a major factor related to the progression of frailty, and UI was recognized as a factor related to prefrail and frail patients. It is necessary to prevent aggravation of the level of frailty through careful assessments and active interventions for nutrition and UI. In addition, since there were some differences in the factors related to deterioration according to frailty level, an individual approach is needed to accommodate each patient’s frailty status.
