# Supplementary material for: Malnutrition as a major related factor of frailty among older adults residing in long-term care facilities in Korea
Source: PLoS One. 2023 Apr 7;18(4):e0283596. doi: 10.1371/journal.pone.0283596 (PMC10081797; doi:10.1371/journal.pone.0283596)
Supplement: Attachment — Submitted filename: Response to Reviewers.docx [file pone.0283596.s001.docx]

| **Response to the Reviewer`s Comments** | |
| --- | --- |
| **Reviewer 1** | **Revision** |
| Thank you for your comments! We have revised the manuscript based on the reviewer’s comments. We also highlighted the revised section in the main text (in blue). | |
| 1. Provide more detail on the study design and sample, including information on sample size calculation and the selection of LTCFs. | Thank you for your comment! We added the information on sample size and selection of LTCFs on page 4-5 |
| 2. Provide more detail on the data collection process, including information on the training of staff who administered the measurements. | Thank you for your comment! We described additional detail information on training of the staffs and data collection process on page 5-6 |
| 3. Describe the statistical analysis in more detail, including information on the variables included in the multivariate analysis and the criteria for determining statistical significance. | Thank you for your comment! We added the more detail information in statistical analysis section on page 8. |
| 4. Provide more context for the results, including a comparison with previous studies on fraily, oral health, nutrition in South Korea such as --  Clin Interv Aging. . 2022 Dec 21;17:1863-1872. PMID: 36575660 PMCID: PMC9790170  BMC Geriatr . 2021 Sep 4;21(1):482. PMID: 34481482 PMCID: PMC8418721  and also discuss the potential implications of the high prevalence of frailty and malnutrition in this population, including the potential impact on morbidity, mortality, and social costs. | Thank you for your very valuable comment! Based on the literature recommended by the reviewer 1, we added discussions on frailty, oral health, and nutrition in S. Korea on page 14, 17-18 .  We also discussed the potential implications of the high prevalence of coexistence of frail and malnutrition on page 15-16. |
| 5. Address any issues with the generalizability of the study, such as the limited number of long-term care facilities and geographic location. | Thank you for your comment. We discussed in the limitation that the generalization of the results may be limited on page 18. |
| 6. Include a discussion on potential interventions for improving the nutritional status of older adults in long-term care facilities and preventing or managing frailty. You may consider to read a Korean study:   Long-term effect of a 24-week multicomponent intervention on physical performance and frailty in community-dwelling older adults. Age Ageing . 2021 Nov 10;50(6):2157-2166. PMID: 34351363 PMCID: PMC8581390 | Thank you for your comment. Based on the literature recommended by the reviewer 1, we further discussed the need for strategic approaches to frailty and malnutrition on page 16. |
| 7. Discuss the potential overlap between the FRAIL-NH and MNA-SF in assessing frailty and malnutrition and how this may affect the results. The use of the FRAIL-NH and MNA-SF to measure frailty and malnutrition, respectively, is appropriate, but it would be helpful to provide more information on the validity and reliability of these instruments in the Korean older adult population.  Ann Geriatr Med Res . 2018 Mar;22(1):20-25. PMID: 32743239 PMCID: PMC7387636 | Thank you for your thoughtful comment! Based on the literature recommended by the reviewer 1, we further discussed the effect of potential overlap in assessing frailty and malnutrition, and the reliability and validity of two instruments on page 15-16. |
| 8. The results section could benefit from more in-depth analysis, including subgroup analysis by sex and age. | Thank you for your great comments! I absolutely agree with your comments for further analysis of frailty differences and predictors according to sex and age groups. However, the comparison by gender was limited to the analysis due to the small sample size for male subgroup. Therefore, this will be considered by adding samples in future studies. In addition, sub-group analysis according to age group is already in progress as another secondary data analysis in our research group. In this study, we intend to proceed with the manuscript with the present result according to the purpose of the study. |
| 9. The language throughout the manuscript could be improved for clarity and conciseness. | Thank you for your comments! This revision of the manuscript has been further edited by the professional native English-speaking editor. |
| 10. Consider including more information on the potential impact of the COVID-19 pandemic on the study. | We added the potential impact of COVID-19 on research in the limitation section on page 19. |
| **Reviewer 2** |  |
| Although the co-existing frailty and malnutrition is well known, the study is one of the rare articles showing this relationship. However, I think references should be updated. In the recent literature showed that there is a close relationship between frailty and nutritional status. And, both MNA-SF and MNA-long form are validated to determine frailty in older patients (<https://pubmed.ncbi.nlm.nih.gov/31118593/>, <https://pubmed.ncbi.nlm.nih.gov/30262439/>) If the authors discuss this issue, it would contribute power for the discussion section. | Thank you for your comments! Based on the literature recommended by the reviewer 1 and 2, we added the discussion the high relationship of the two instruments on page 15. |
